# Smartphone and medical application use among dentists in China

**DOI:** 10.1186/s12911-020-01238-3

**Published:** 2020-09-07

**Authors:** Chao Zhang, Lin Fan, Zhaowu Chai, Cong Yu, Jinlin Song

**Affiliations:** 1 College of Stomatology, Chongqing Medical University, Chongqing, China; 2 Chongqing Key Laboratory for Oral Diseases and Biomedical Sciences, Chongqing, China; 3 Chongqing Municipal Key Laboratory of Oral Biomedical Engineering of Higher Education, Chongqing, China

**Keywords:** Smartphone, Medical applications, Dentistry, China, Dentists

## Abstract

**Background:**

With the development of information technology, an increasing number of healthcare professionals are using smartphones and mobile medical applications (apps) in their clinical practice. The objective of this study was to survey the use of smartphone-based medical apps among dentists in China and determine dentists’ perceptions of such apps.

**Methods:**

All data were collected using anonymous questionnaires. The questionnaires for this cross-sectional study were randomly sent to dentists by email, and 379 dentists responded. Dentists’ demographics and perceptions of WeChat, QQ (the most popular social media apps in China) and other medical apps were assessed; the questionnaire including questions on the purpose, frequency, daily use, and opinion of the apps they used. Questions were answered using a Likert scale (1 = strongly agree, 2 = agree, 3 = not sure, 4 = disagree, and 5 = strongly disagree).

**Results:**

A total of 379 valid responses were received; the respondents had a median age of 33.6 years old (63.3% female). All subjects (100%) owned a smartphone, and all of them installed and used WeChat or QQ in their clinical practice. Only 76% of subjects installed medical apps (other than WeChat and QQ) on their smartphones. Male dentists were more likely to install medical apps than female dentists (*p* < 0.05). With increasing age, the percentage of dentists who installed medical apps decreased (*p* < 0.001). The frequency and daily use were higher for WeChat and QQ than for medical apps. Medical apps were positively perceived, with dentists reporting that they recommend these medical apps to their peers (Likert score: 1.67 ± 0.68).

**Conclusion:**

Medical apps were perceived to have a positive impact on clinical practice, education and patient care in dentistry by providing relevant medical information. However, there will still be much room for improvement in the future.

## Background

With the development and decreasing costs of information technology, an increasing number of people are becoming internet users worldwide. Data show that the number of internet users in the world was nearly 4,536,248,808 by June 30, 2019 [1]. There are 2,300,469,859 internet users in Asia, representing 54.2% of the world. To date, mobile devices (such as mobile smartphones and tablets) are the main tools for accessing the internet. Smartphones have become handheld computers rather than mobile communication devices because of their powerful computing function, spacious memories, large screens and open operating systems. The tremendous improvements of smartphones has encouraged the development of various third-party applications (apps) that can be used on smartphones [2–5]. An app is a small specialized and customizable program that can provide various functions and services for users. There are numerous apps, including medical apps, available for use on smartphones. In addition to the general public, an increasing number of healthcare professionals are using medical apps in their clinical practice [2]. In the current information society, medicine has undergone remarkable advancements. Many studies have surveyed the use of smart devices or medical applications among physicians [6–8]. In dental fields, we only found similar studies on the use of medical applications or mobile learning technology among dental students around the world [9–12]. However, such research in dentists is limited, especially in China.

From the data available regarding the number of internet users worldwide it is reasonable to assume that Asia (especially China) will be a large market for smartphones and applications. Therefore, the aim of our study was to examine the current use of smartphone-based medical apps among dentists in China and determine the dentists’ attitudes toward these apps. We aimed to provide readers with a better understanding of (1) the popularity of smartphones and medical apps among dentists in China, (2) the factors influencing dentists’ use of medical apps, and (3) dentists’ purposes for using such medical apps and attitudes towards them. With the data from this study, we hope computer scientists or dental healthcare professionals can design, improve and develop more convenient and useful smartphone-based applications according to current dentists’ opinions. We also hope to help the dentists who have not used medical apps to select the proper apps to assist their clinical work.

### Related work

Many studies conducted surveys regarding the use of smart devices or medical applications among physicians or medical students (Table 1). The aim of this study was to survey the use of smartphone-based medical apps among dentists in China and determine the dentists’ perceptions of such apps.

**Table 1:** Previous works

Name	Research object	Country	Objective	Result
Orrin et al. (2012) [8]	Medical providers	USA	The use of smartphones and smartphone apps among providers at medical centers recognized by the Accreditation Council for Graduate Medical Education (ACGME)	1. A total of 3306 unique responses from 1397 residents, 524 fellows, and 1385 attending physicians among 27 different specialties attended the Study. 2. Greater than 85% of respondents used a smartphone. 3. Over half of the respondents reported using apps in their clinical practice. 4. the most commonly used app types were drug guides, medical calculators, coding and billing apps and pregnancy wheels.
Karl et al. (2012) [11]	Medical students, junior doctors	United Kingdom	Smartphone and medical related App use among medical students and junior doctors	1. 257 medical students and 131 junior doctors attended the study. 2. 79.0% of medical students and 74.8% of junior doctors owned a smartphone. 3. The majority of students and doctors owned 1–5 medical related applications, with very few owning more than 10. 4. Over 24 h apps were used for between 1 and 30 min for students and 1–20 min for doctors. 5. Students used disease diagnosis/management and drug reference apps, with doctors favouring clinical score/calculator apps.
Rikesh et al. (2015) [7]	Surgical doctors	United Kingdom	Smartphone use amongst doctors within the surgical profession	1. A total of 341 participants were surveyed. 2. 93.5% of which owned a smartphone, with 54.2% of those owning medical apps and 86.2% using their device to access online medical resources. 3. 79.3% stated that they would be willing to use their smartphone for clinical use.
Payal et al. (2018) [13]	Dental students	Central Indian	Digital literacy and use of smart phones among Central Indian dental students	1. Out of 260 students, 250 were internet users. 2. 94.23% students owned a smartphone. 3. 46.53% students had some app related to the dentistry in their smartphone device. 4. Nearly 80% dental students believed that social media helps them in their professional course studies. 5. 89.23% students were keen for implementation of e-learning in their curriculum.
Sameer et al. (2018) [14]	Physicians	Saudi Arabia	Popularity and impact of using smart devices in medicine in Saudi Arabia	1. 300 physicians attended the study. 2. 88.3% physicians had smart devices, and 86.3% had at least one medical app installed. 3. 53.0% used an app at least once a day. 4. Medical apps were positively perceived, with physicians reporting increased dependency on the apps

In 2012, Orrin et al. performed a prospective, nationwide email survey evaluating the use of smartphones and smartphone apps among providers at medical centers recognized by the Accreditation Council for Graduate Medical Education (ACGME) in the United States of America (USA) [8]. A total of 3306 unique responses from 1397 residents, 524 fellows, and 1385 attending physicians were received in the study. Greater than 85% of respondents used a smartphone. Over half of the respondents reported using apps in their clinical practice. The most commonly used app types were drug guides, medical calculators, coding and billing apps and pregnancy wheels.

Karl et al. conducted an online survey on smartphone- and medical-related app use among medical students and junior doctors in the United Kingdom (UK) in 2012 [11]. A total of 79.0% of medical students and 74.8% of junior doctors owned a smartphone. On average, apps were used for between 1 and 30 min per 24 h for students and 1–20 min per 24 h for doctors.

In 2018, Rikesh et al. assessed smartphone use among UK surgical doctors [7]. A total of 341 participants were surveyed. A total of 93.5% of participants owned a smartphone, with 54.2% of those owning medical apps and 86.2% using their device to access online medical resources. A total of 79.3% stated that they would be willing to use their smartphone for clinical use.

Payal et al. performed an assessment of digital literacy and the use of smart phones among Central Indian dental students [13]. Out of 260 students, 250 were internet users. A total of 94.23% of students owned a smartphone, and 46.53% of students had at least one app related to dentistry on their smartphone device. A total of 89.23% of students were keen for the implementation of e-learning in their curriculum.

## Methods

### Questionnaire and procedures

Considering that WeChat and QQ are the most popular social media apps in China, we investigated their use in dental clinical practice separately. The medical apps investigated in our study were a series of apps that are associated with clinical practice (other than WeChat and QQ).

The study questionnaire consisted of several parts (supplementary files). Part 1 collected dentists’ demographic data, including age, gender, workplace, medical rank, whether a smartphone was used, and brand of smartphone. Part 2 investigated the use of WeChat and QQ in dentists’ clinical work, including whether or not they used WeChat and QQ for work-related activities, and the purpose, frequency and daily use time of these apps. Part 3 surveyed the use of medical apps in dentists’ clinical work, including whether medical apps were installed, how many apps were installed, and the purpose, frequency and daily use time of these medical apps. Part 4 assessed the dentists’ perceptions of smartphone-based medical apps. Part 5 assessed the impact of medical apps on clinical practice. Responses to questions in parts 4–5 were based on a 5-point Likert scale: 1 = strongly agree, 2 = agree, 3 = not sure, 4 = disagree, and 5 = strongly disagree.

**Fig. 1:**
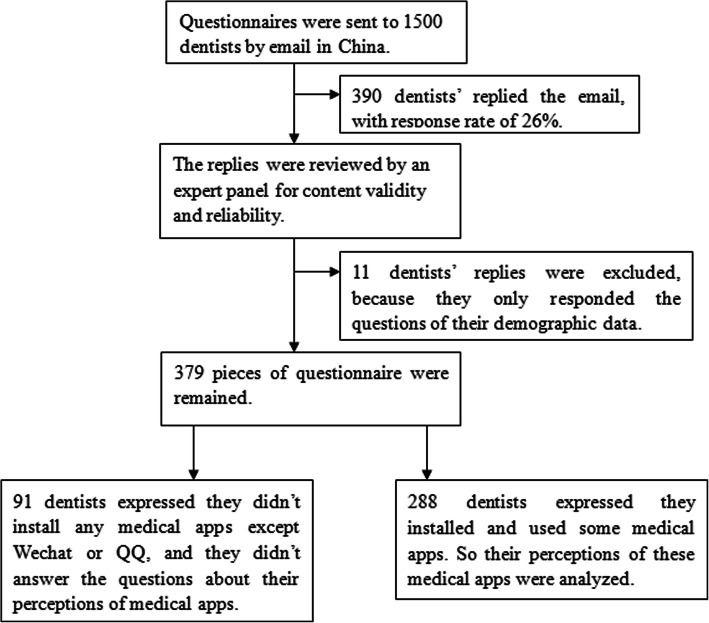
Trial Flow Diagram. This figure presents the trial flow diagram of collecting eligible questionnaires for this study

The questionnaire was sent to 1500 dentists by email; we received 390 replies (response rate of 26%). The questionnaire was reviewed by an expert panel for content validity and reliability. Eleven dentists only responded to the questions in part 1, so we did not include their questionnaires in the analysis. Of the remaining 379 subjects, 91 dentists expressed that they did not install any medical apps except WeChat or QQ, and they did not answer the questions in part 4 and part 5. Therefore, we only analyzed the remaining 288 dentists’ perceptions of these medical apps. The flow diagram outlining the collection of eligible questionnaires for this study is shown in Fig. 1.

### Statistical analysis

Data were analyzed using SPSS statistical software (version 17, SPSS, Inc., Chicago, IL, USA). Subject characteristics are presented as frequencies (percentages). Quantitative Likert scale data are presented as the mean ± standard deviation. A chi-square test was used to examine the difference in owning a medical app among different groups. A *p* value of < 0.05 was considered significant.

## Results

### Demographic characteristics of surveyed dentists in China

A total of 379 valid responses were received. The median study subject age was 33.6 years old (range: 19–59 years old), and 240 (63.3%) subjects were female. A total of 168 (44.3%) subjects worked in public hospitals, and 211 (55.7) subjects worked in private hospitals or clinics. Additionally, 47.2% of subjects were resident dentists, 40.4% were dentists in charge, and 12.4% were senior or associate senior dentists. All subjects (100%) owned a smartphone. The most popular brands of smartphones were Apple (46.2%) and Huawei (34%, Table 2).

**Table 2:** Demographic characteristics of surveyed dentists in China (*n* = 379 subjects)

	Number	Percent (%)
Age		
≤ 30	152	40.1
31–40	181	47.8
41–50	24	6.3
> 50	22	5.8
Gender		
Male	139	36.7
Female	240	63.3
Workplace		
Public hospital	168	44.3
Private hospital or clinic	211	55.7
Medical rank		
Resident dentist	179	47.2
Dentist in charge	153	40.4
Senior/associate senior dentist	47	12.4
Do you own a smartphone?		
Yes	379	100
What’s the brand of your smarphone?		
Apple	175	46.2
Huawei	129	34
OPPO	33	8.7
Xiaomi	28	7.4
Sumsung	6	1.6
Others	8	2.1

### The use of WeChat or QQ in dentists’ clinical work

All participants installed WeChat or QQ on their smartphones, and they all used WeChat or QQ in their clinical practices. Participants reported that their main purposes for using WeChat or QQ in clinical practice were acquiring medical information (84.2%), communicating with peers (83.6%) and communicating with patients (77.6%). The frequency of using WeChat or QQ in clinical practice was reported to be at least once a day (73.6%), at least once a week (20.8%), or less than once a month (5.5%, Table 3). The time of daily usage is shown in Table 3; 25.9% subjects used WeChat or QQ in their clinical practice for more than 60 min per day, 19% subjects reported their daily use time was 21–30 min, 17.4% subjects reported their daily use time was 11–20 min.

**Table 3:** The use of Wechat or QQ in dentists’ clinical work (n = 379 subjects)

	Number	Percent (%)
Have you installed Wechat or QQ on your smartphone?		
Yes	379	100
Do you use WeChat or QQ in clinical practice?		
Yes	379	100
The purpose for using WeChat or QQ in clinical practice		
Communicating with patients	294	77.6
Communicating with peers	317	83.6
Acquiring medical information	319	84.2
Others	76	20
Frequency of using WeChat or QQ in clinical practice		
At least once a day	279	73.6
At least once a week	79	20.8
Less than once a month	21	5.5
Daily use of WeChat or QQ in clinical practice within dentists (in minutes)		
None	0	0
1–10 min	51	13.5
11–20 min	66	17.4
21–30 min	72	19
31–40 min	49	12.9
41–50 min	24	6.3
51–60 min	19	5.0
> 60 min	98	25.9

### The use of medical apps in dentists’ clinical work

A total of 288 (76%) subjects had medical apps (other than WeChat and QQ) installed on their smartphones, and 91 (24%) subjects did not have any medical apps installed, as shown in Table 4. Male dentists were more likely to install medical apps than female dentists (chi-square = 6.702, *p* < 0.05). With increasing age, the percentage of dentists who had medical apps installed decreased (chi-square = 45.3.3, *p* < 0.001). However, dentists’ workplace and medical rank were not significantly associated with the installation of medical apps (chi-square = 1.670, *p* = 0.196; chi-square = 5.466, *p* = 0.065, respectively, Table 5). Among the 288 subjects, the average number of medical apps installed was 2.62 ± 1.73 (range: 1–12). Most subjects (71.5%) reported that they had 1–5 medical apps installed on their smartphones, and only 0.5% of subjects had more than 10 medical apps installed on their smartphones (Table 4). The frequency and daily use (in minutes) of these medical apps among dentists is shown in Table 6. A total of 46.5% of subjects used medical apps at least once a day, 42.4% of subjects used them at least once a week, and 11.1% used them less than once a month. Among those who reported daily use, most reported 11–20 min of usage (31.6%) per day, followed by 11–10 min (29.2%) and 21–30 min (15.6%). Only 5.2% of subjects reported that they used medical apps more than 60 min per day (Table 6). The top three purposes for using medical apps were reviewing medical knowledge (63.6%), reading medical news (36.4%), and reading medical journals (27.9%, Table 7).

**Table 4:** The use of medical apps in dentists’ clinical work (n = 379 subjects)

	Number	Percent (%)
Have you installed medical apps on your smartphone? (except Wechat and QQ)		
Yes	288	76
No	91	24
How many medical apps do you have on your smartphone?		
No	91	24
1–5 apps	271	71.5
6–10 apps	15	4
> 10 apps	2	0.5

**Table 5:** The factors of affecting whether or not dentists installed medical apps

	Whether or not dentist installed medical apps		Total	Chi-square test	df	*p*-value
	Yes	No				
Gender						
Male	116	23	139	6.702	1	0.010
Female	172	68	240			
Age						
≤ 30	124	28	152	45.303	3	0.000
31–40	144	37	181			
41–50	16	8	24			
> 50	4	18	22			
Workplace						
Public hospital	133	35	168	1.670	1	0.196
Private hospital or clinic	155	56	211			
Medical rank						
Resident dentist	135	44	179	5.466	2	0.065
Dentist in charge	123	30	153			
Senior/associate senior dentist	30	17	47			
Total	288	91	379

**Table 6:** The frequency and daily use (in minutes) of medical apps within dentists in China (*n* = 288 subjects)

	Number	Percent (%)
Frequency		
At least once a day	134	46.5
At least once a week	122	42.4
Less than once a month	32	11.1
Daily use		
None	2	0.7
1–10 min	84	29.2
11–20 min	91	31.6
21–30 min	45	15.6
31–40 min	30	10.4
41–50 min	16	5.6
51–60 min	5	1.7
> 60 min	15	5.2

**Table 7:** Purpose for using smartphone based medical apps (*n* = 288 subjects)

	Number	Percent (%)
Reviewing medical knowledge	241	63.6
Reading medical news	138	36.4
Reading medical journals	106	27.9
Clinical skills guide	105	27.7
Communicating with patients	98	25.9
Communicating with colleages	97	25.6
Preparing presentations	87	22.9
Assisting diagnosis	80	21.1
Exam preparation	67	17.7
During ward rounds	64	16.9
Medication or drug guide	64	16.9
Scheduling patients’ appointment	47	12.4
Mobile learning	30	7.9

### Perceptions of smartphone-based medical apps and their impacts on clinical practice

The majority of subjects strongly agreed or agreed that they are looking to obtain more medical apps in the future (1.52 ± 0.69), that they would recommend these medical apps to other peers (1.67 ± 0.68), that medical apps are essential tools for undergraduate medical studies (1.91 ± 0.78), and that medical apps supplement medical textbooks (1.83 ± 0.64). Regarding whether medical apps are superior to medical textbooks (3.16 ± 0.81) or whether medical apps can replace medical textbooks (3.31 ± 0.94), the majority of subjects reported disagreement or that they were not sure. A total of 46.2% of subjects agreed that there are dangers in using medical apps for patient care, and 39.6% of subjects were not sure about this question (2.61 ± 0.73, Table 8).

**Table 8:** Dentists’ perceptions on smartphone based medical apps (*n* = 288 subjects)

		Number	Percent (%)	Mean	SD
Medical apps are easy to obtain	Strongly agree	56	19.4	2.36	0.99
	Agree	127	44.1		
	Not sure	51	17.7		
	Disagree	54	18.8		
	Stongly disagree	0	0		
I am looking to obtain more medical apps in the future	Strongly agree	160	55.6	1.52	0.69
	Agree	114	39.6		
	Not sure	5	1.7		
	Disagree	9	3.1		
	Stongly disagree	0	0		
I would recommend these medical apps to other peers	Strongly agree	122	42.4	1.67	0.68
	Agree	148	51.4		
	Not sure	10	3.5		
	Disagree	8	2.8		
	Stongly disagree	0	0		
I do most of my medical learning using medical apps	Strongly agree	62	21.5	2.43	1.17
	Agree	131	45.5		
	Not sure	9	3.1		
	Disagree	80	27.8		
	Stongly disagree	6	2.1		
Medical apps are essential tools for undergraduate medical studies	Strongly agree	91	31.6	1.91	0.78
	Agree	140	48.6		
	Not sure	48	16.7		
	Disagree	9	3.1		
	Stongly disagree	0	0		
Medical apps are superior to medical textbooks	Strongly agree	13	4.5	3.16	0.81
	Agree	56	19.4		
	Not sure	98	34		
	Disagree	115	39.9		
	Stongly disagree	6	2.1		
Medical apps can replace medical textbooks	Strongly agree	7	2.4	3.31	0.94
	Agree	68	23.6		
	Not sure	48	16.7		
	Disagree	159	55.2		
	Stongly disagree	6	2.1		
Medical apps supplement medical textbooks	Strongly agree	77	26.7	1.83	0.64
	Agree	193	67		
	Not sure	9	3.1		
	Disagree	8	2.8		
	Stongly disagree	1	0.3		
Medical apps provide useful point-of-care medical information	Strongly agree	54	18.8	2.28	0.97
	Agree	149	51.7		
	Not sure	34	11.8		
	Disagree	51	17.7		
	Stongly disagree	0	0		
There are dangers in using medical apps for patient care	Strongly agree	7	2.4	2.61	0.73
	Agree	133	46.2		
	Not sure	114	39.6		
	Disagree	34	11.8		
	Stongly disagree	0	0		

The majority of subjects agreed that medical apps could improve clinical decision making (2.30 ± 0.77), save time (2.11 ± 0.68), help in making differential diagnoses (2.56 ± 0.74), and perform useful medical-related calculations (2.52 ± 0.77). Additionally, medical apps were thought to be beneficial for allowing faster access to evidence-based medical practices/cases (2.34 ± 0.80); they were reported to be reliable sources of clinical skills (2.25 ± 0.67), medical knowledge (2.19 ± 0.68), common laboratory reference values (2.30 ± 0.65), and medical information (1.90 ± 0.54, Table 9).

**Table 9:** Perceived impact of smartphone based medical apps on clinical practice

		Number	Percent (%)	Mean	SD
Improve clinical decision-making	Strongly agree	30	10.4	2.30	0.77
	Agree	166	57.6		
	Not sure	67	23.3		
	Disagree	25	8.7		
	Stongly disagree	0	0		
Save time	Strongly agree	41	14.2	2.11	0.68
	Agree	185	64.2		
	Not sure	51	17.7		
	Disagree	11	3.8		
	Stongly disagree	0	0		
Allow faster access to medical information	Strongly agree	53	18.4	1.90	0.54
	Agree	214	74.3		
	Not sure	17	5.9		
	Disagree	4	1.4		
	Stongly disagree	0	0		
Allow faster access to common laboratory reference values	Strongly agree	16	5.6	2.30	0.65
	Agree	185	64.2		
	Not sure	72	25		
	Disagree	15	5.2		
	Stongly disagree	0	0		
Help in developing differential diagnoses	Strongly agree	7	2.4	2.56	0.74
	Agree	176	61.1		
	Not sure	70	24.3		
	Disagree	35	12.2		
	Stongly disagree	0	0		
Perform useful medical related calculations	Strongly agree	8	2.8	2.52	0.77
	Agree	163	56.6		
	Not sure	78	27.1		
	Disagree	38	13.2		
	Stongly disagree	1	0.3		
Allow faster access to reliable sources of medical knowledge	Strongly agree	28	9.7	2.19	0.68
	Agree	194	67.4		
	Not sure	50	17.4		
	Disagree	16	5.6		
	Stongly disagree	0	0		
Allow faster access to reliable sources of clinical skills	Strongly agree	24	8.3	2.25	0.67
	Agree	180	62.5		
	Not sure	71	24.7		
	Disagree	13	4.5		
	Stongly disagree	0	0		
Allow faster access to evidence-based medical practice/case	Strongly agree	39	13.5	2.34	0.80
	Agree	132	45.8		
	Not sure	96	33.3		
	Disagree	21	7.3		
	Stongly disagree	0	0		

## Discussion

In recent decades, the technologies of mobile communication, mobile wireless internet and mobile devices have been widely used in many areas of human life. In this context, customized and multifunctional mobile apps (including medical apps) have flourished tremendously. Studies have shown that an increasing number of medical staff installed medical apps on their smart devices (including smartphones and tablets) and used them frequently [2]. However, few studies have assessed the use of such medical applications among dentists. Therefore, in this study, we first performed such research in China. By using anonymous questionnaires, we collected data on the use of smartphone-based medical apps among dentists in China.

We found that all participants (100%) in our study owned a smartphone, and the rate was slightly higher than many other similar studies (ranging from 82 to 99.3%) [14–18]. The most popular brands of smartphones were Apple and Huawei (a Chinese Android smartphone), and this result was consistent with another study [18]. All subjects had WeChat or QQ (the most popular social media applications in China) installed on their smartphones and used them in clinical practice. In 2016, Li et al. investigated the effect of WeChat on the compliance and duration of treatment in orthodontic patients in China. The authors found that use of this app could reduce the treatment duration and bracket bond failure and improve clinic attendance in orthodontic patients [19]. Additionally, in our study, we found that 73.6% of subjects used these apps at least once a day, and only 5.5% of subjects used them less than once a month. A total of 25.9% of subjects reported that they typically used them in clinical practice more than 60 min per day. As we can see, the use of such social media apps (for purposed including acquiring medical information, communicating with peers and communicating with patients) is very common among dentists in China.

Only 76% of the participants (*n* = 288) installed a medical app (other than WeChat and QQ) on their smartphones. Though the rate was lower compared with the installation of WeChat and QQ, the number was still in agreement with many studies in other areas of the world [14, 15, 20]. Our study found that there were significant differences in whether medical apps were installed by gender and age. This finding was supported by many studies, in which young physicians were more likely to use medical apps than old physicians [7, 14]. In our study, male dentists were more likely to use medical apps than females. Males are more interested in Internet technology and software in China, which could explain this observed phenomenon.

The average number of installed medical apps was 2.62, which was relatively small. According to the data regarding frequency and daily use of these medical apps, only 46.5% of subjects used medical apps at least once a day, and only 5.2% of subjects reported that they used medical apps more than 60 min per day. Compared with the use of WeChat or QQ in China, we found that the use of medical apps among dentists in China was not very prevalent. Compared with US and British healthcare professionals with a use rate of over 90%, our sample of dentists still showed a relatively lower medical app use [21, 22].

Most of our subjects agreed or strongly agreed that they are looking to obtain more medical apps in the future and recommend these medical apps to other peers, which could reflect their affirmation and expectation towards medical apps. Most subjects agreed or strongly agreed that medical apps are essential tools for undergraduate medical studies and supplement medical textbooks. These findings were in agreement with several other studies that assessed the use of medical apps among dental students or junior doctors in many other regions around the world [9–12, 23–27]. Although dentists thought medical apps could improve education among students, they reported disagreement or uncertainty with regard to whether medical apps are superior to medical textbooks or medical apps can replace medical textbooks. Most of them agreed that there are dangers in using medical apps for patient care. As we know, the accuracy of the information in a medical app is very important. If users make their clinical decision based on inaccurate or outdated information in medical apps, there could be serious consequences. Many studies have been conducted to assess the quality and accuracy (including expert involvement and medical evidence adherence) of these medical applications in the past few years [28–34]. The expert involvement rate of these applications ranged from 9 to 67%, and the adherence rate ranged from 0 to 87% [28]. Therefore, establishing appropriate regulatory procedures is extremely urgent. We believe that government health authorities (such as the Food and Drug Administration in America [35], the Medicines and Healthcare Products Regulatory Agency in England [36], Health Canada in Canada [37] and corresponding authorizes in other countries) could play a key role. To regulate these medical apps well, government health authorities could draft relevant guidelines that should be followed by app developers.

Regarding the effect of medical apps on clinical practice, dentists in China strongly agreed or agreed that these medical apps could allow faster access to medical information (1.90 ± 0.54). However, regarding the other aspects of the effect, Chinese dentists’ attitudes were less positive than those reported in a similar study [14]. From the results above, we found that current medical apps in dentistry did well in providing relevant medical information in China and received positive reviews from Chinese dentists. However, on the other hand (i.e. outside of improved clinical decision making; saving time; help in making differential diagnoses; performing useful medical-related calculations; faster access to evidence-based medical practices/cases; and providing reliable sources of clinical skills, knowledge, and common laboratory reference values), medical apps did not meet the needs of dentists well. Therefore, in the future, there will be much room for improvement.

## Conclusion

In conclusion, the use of smartphones and some social media apps (WeChat or QQ) is very common among dentists in China. The use of medical apps is also prevalent. These medical apps received positive reviews because most dentists reported that they would want to obtain more medical apps in the future and recommend these medical apps to other peers. These medical apps could allow dentists faster access to medical information in their clinical practices. However, there is still much room for improvement in patient care in the future (such as assisting with diagnoses and determining treatment options).

According to this study, we found that there are many advantages of medical apps, including reviewing medical knowledge, reading medical or journals, acquiring clinical skills, communicating with patients/colleagues, and preparing presentations or examinations. In general, medical apps could be a good assistant for dentists in clinical practice. We believe that there will be an increasing number of dentists using medical apps in the future. Of course, patients would also benefit from these apps. However, there are still some disadvantages or risks of medical apps, such as data security, virus attack, inaccurate content, and lack expert involvement, to which we should pay attention. Therefore, establishing appropriate regulatory procedures is extremely important. We think the following tips could help [38]. First, the app stores should carefully examine any medical apps that would like to be published online. Second, the content and information in medical apps should be peer reviewed by relevant medical professionals. Third, a reliable assessment system or method for these medical applications should be established. An assessment or score of medical apps could help users select more appropriate apps for their purposes. Finally, we think the involvement of government health authorities is most important. We believe that the risks of using medical apps could be reduced by following these methods.

## Limitations

There were some limitations in our study. First, our sample size was relatively small. We need a larger sample size to confirm our findings on medical app use among dentists in China in the future. Nevertheless, our results are in agreement with many similar studies conducted in other regions around the world and provide preliminary information. Second, our study only examined dentists’ perceptions of medical apps. Further studies should be conducted to examine dental patients’ use and perceptions of medical apps to examine how medical apps affect their oral healthcare. Third, our study did not investigate the potentially negative impacts of medical app use. Because the issue of patient safety and privacy has been proposed in recent years, we should focus on these issues in further investigations.

## Supplementary information

**Additional file 1.**

**Additional file 2.**

**Additional file 3.**

## Data Availability

You could find the date in additional supporting files.

## Abbreviations

Apps: Applications
ACGME: Accreditation Council for Graduate Medical Education
USA: United States of America
UK: United Kingdom

## References

[1] Internet Word Stats. World Internet Users and 2019 Population Stats. [https://www.internetworldstats.com/stats.htm]. Accessed 2 Jan 2020.

[2] Mosa AS, Yoo I, Sheets L (2012). A systematic review of healthcare applications for smartphones. BMC Med Inform Decis Mak..

[3] Boulos MN, Wheeler S, Tavares C (2011). How smartphones are changing the face of mobile and participatory healthcare: an overview, with example from eCAALYX. Biomed Eng Online.

[4] Baheti MJ, Toshniwal N (2014). Orthodontic apps at fingertips. Prog Orthod.

[5] Franko OI (2011). Smartphone apps for orthopaedic surgeons. Clin Orthop Relat Res.

[6] Ozdalga E, Ozdalga A, Ahuja N (2012). The smartphone in medicine: a review of current and potential use among physicians and students. J Med Internet Res.

[7] Patel RK, Sayers AE, Patrick NL (2015). A UK perspective on smartphone use amongst doctors within the surgical profession. Ann Med Surg (Lond).

[8] Franko OI, Tirrell TF (2012). Smartphone app use among medical providers in ACGME training programs. J Med Syst.

[9] Khatoon B, Hill KB, Walmsley AD (2014). Dental students’ uptake of mobile technologies. Br Dent J.

[10] Suner A, Yilmaz Y, Piskin B (2019). Mobile learning in dentistry: usage habits, attitudes and perceptions of undergraduate students. PeerJ..

[11] Payne KB, Wharrad H, Watts K (2012). Smartphone and medical related app use among medical students and junior doctors in the United Kingdom (UK): a regional survey. BMC Med Inform Decis Mak.

[12] Masika MM, Omondi GB, Natembeya DS (2015). Use of mobile learning technology among final year medical students in Kenya. Pan Afr Med J.

[13] Saxena P, Gupta SK, Mehrotra D (2018). Assessment of digital literacy and use of smart phones among central Indian dental students. J Oral Biol Craniofac Res.

[14] Al-Ghamdi S (2018). Popularity and impact of using smart devices in medicine: experiences in Saudi Arabia. BMC Public Health.

[15] Jahanshir A, Karimialavijeh E, Sheikh H (2017). Smartphones and medical applications in the emergency department daily practice. Emerg (Tehran).

[16] Illiger K, Hupka M, von Jan U (2014). Mobile technologies: expectancy, usage, and acceptance of clinical staff and patients at a university medical center. JMIR Mhealth Uhealth..

[17] Grow JN, Vargo JD, Nazir N (2019). Smartphone applications in plastic surgery: a cross-sectional survey of 577 plastic surgeons, fellows, residents, and medical students. Aesthet Surg J.

[18] Shaw H, Ellis DA, Kendrick LR (2016). Predicting smartphone operating system from personality and individual differences. Cyberpsychol Behav Soc Netw.

[19] Li X, Xu ZR, Tang N (2016). Effect of intervention using a messaging app on compliance and duration of treatment in orthodontic patients. Clin Oral Investig.

[20] Hofer F, Haluza D (2019). Are Austrian practitioners ready to use medical apps? Results of a validation study. BMC Med Inform Decis Mak..

[21] Johnson AC, El Hajj SC, Perret JN (2015). Smartphones in medicine: emerging practices in an academic medical center. J Med Syst.

[22] Mark D, Leonard C, Breen H (2014). Mobile phones in clinical practice: reducing the risk of bacterial contamination. Int J Clin Pract.

[23] Rung A, Warnke F, Mattheos N (2014). Investigating the use of smartphones for learning purposes by Australian dental students. JMIR Mhealth Uhealth..

[24] Bullock A, Dimond R, Webb K (2015). How a mobile app supports the learning and practice of newly qualified doctors in the UK: an intervention study. BMC Med Educ.

[25] Cox S, Pollock D, Rountree J (2016). Use of information and communication technology amongst New Zealand dental students. Eur J Dent Educ.

[26] Greene LR, Spuur KM (2018). Undergraduate use of medical radiation science mobile applications. Radiography (Lond).

[27] Scarbecz M, DeSchepper EJ (2018). Trends in first-year dental Students’ information technology knowledge and use: results from a U.S. dental school in 2009, 2012, and 2017. J Dent Educ.

[28] Subhi Y, Bube SH, Rolskov Bojsen S (2015). Expert involvement and adherence to medical evidence in medical Mobile phone apps: a systematic review. JMIR Mhealth Uhealth..

[29] Buijink AW, Visser BJ, Marshall L (2013). Medical apps for smartphones: lack of evidence undermines quality and safety. Evid Based Med.

[30] Breland JY, Yeh VM, Yu J (2013). Adherence to evidence-based guidelines among diabetes self-management apps. Transl Behav Med.

[31] Abroms LC, Lee Westmaas J, Bontemps-Jones J (2013). A content analysis of popular smartphone apps for smoking cessation. Am J Prev Med.

[32] Pagoto SL, Schneider KL, Jojic M (2014). Weight loss using evidence-based strategies in mobile apps. Am J Prev Med.

[33] Breton ER, Fuemmeler BF, Abroms LC (2011). Weight loss-there is an app for that! But does it adhere to evidence-informed practices?. Transl Behav Med.

[34] Mobasheri MH, Johnston M, King D (2014). Smartphone breast applications – what’s the evidence?. Breast..

[35] Food and Drug Administration. Policy for Device Software Functions and Mobile Medical Applications. Guidance for Industry and Food and Drug Administration Staff. [https://www.fda.gov/regulatory-information/search-fda-guidance-documents/policy-device-software-functions-and-mobile-medical-applications]. Accessed 2 Jan 2020.

[36] Boulos MN, Brewer AC, Karimkhani C (2014). Mobile medical and health apps: state of the art, concerns, regulatory control and certification. Online J Public Health Inform.

[37] Zawati MH, Lang M (2019). Mind the app: considerations for the future of Mobile health in Canada. JMIR Mhealth Uhealth.

[38] Lewis TL, Wyatt JC (2014). mHealth and mobile medical apps: a framework to assess risk and promote safer use. J Med Internet Res.

