# Supplementary material for: Smartphone and medical application use among dentists in China
Source: BMC Med Inform Decis Mak. 2020 Sep 7;20:213. doi: 10.1186/s12911-020-01238-3 (PMC7487503; doi:10.1186/s12911-020-01238-3)
Supplement: Supplementary file 3 — Additional file 3. [file 12911_2020_1238_MOESM3_ESM.doc]

The questionnaires.

Part 1

| Age |  |
| --- | --- |
| Gender |  |
| Workplace | Public hospital  Private hospital or clinic |
| Medical rank | Resident dentist  Dentist in charge  Senior/associate senior dentist |
| Do you own a smartphone? | Yes  No |
| The brand of your smartphone |  |

Part 2

| Have you installed Wechat or QQ on your smartphone? | Yes  No |
| --- | --- |
| Do you use WeChat or QQ in clinical practice? | Yes  No |
| The purpose for using WeChat or QQ in clinical practice | Communicating with patients  Communicating with peers  Acquiring medical information  Others |
| Frequency of using WeChat or QQ in clinical practice | At least once a day  At least once a week  Less than once a month |
| Daily use of WeChat or QQ in clinical practice within dentists (in minutes) | None  1-10 minutes  11-20 minutes  21-30 minutes  31-40 minutes  41-50 minutes  51-60 minutes  >60 minutes |

Part 3

| Have you installed medical apps on your smartphone? (except Wechat and QQ) | Yes  No |
| --- | --- |
| How many medical apps do you have on your smartphone? |  |
| The purpose for using smartphone based medical apps |  |
| Frequency of using medical apps in clinical practice | At least once a day  At least once a week  Less than once a month |
| Daily use of medical apps in clinical practice within dentists (in minutes) | None  1-10 minutes  11-20 minutes  21-30 minutes  31-40 minutes  41-50 minutes  51-60 minutes  >60 minutes |

Part 4

| Medical apps are easy to obtain | Strongly agree  Agree  Not sure  Disagree  Strongly disagree |
| --- | --- |
| I am looking to obtain more medical apps in the future |  |
| I would recommend these medical apps to other peers |  |
| I do most of my medical learning using medical apps |  |
| Medical apps are essential tools for undergraduate medical studies |  |
| Medical apps are superior to medical textbooks |  |
| Medical apps can replace medical textbooks |  |
| Medical apps supplement medical textbooks |  |
| Medical apps provide useful point-of-care medical information |  |
| There are dangers in using medical apps for patient care |  |

Part 5

| Improve clinical decision-making | Strongly agree  Agree  Not sure  Disagree  Strongly disagree |
| --- | --- |
| Save time |  |
| Allow faster access to medical information |  |
| Allow faster access to common laboratory reference values |  |
| Help in developing differential diagnoses |  |
| Perform useful medical related calculations |  |
| Allow faster access to reliable sources of medical knowledge |  |
| Allow faster access to reliable sources of clinical skills |  |
| Allow faster access to evidence-based medical practice/case |  |
